# Predictive value of soluble ST2 in adolescent and adult patients with complex congenital heart disease

**DOI:** 10.1371/journal.pone.0202406

**Published:** 2018-08-17

**Authors:** Mohammed Laqqan, Christiane Schwaighofer, Stefan Graeber, Tanja Raedle-Hurst

**Affiliations:** 1 Department of Gynecology and Obstetrics, Saarland University Medical Center, Homburg/Saar, Germany; 2 Department of Pediatric Cardiology, Saarland University Medical Center, Homburg/Saar, Germany; 3 Institute of Medical Biometry, Epidemiology and Medical Informatics, Saarland University Medical Center, Homburg/Saar, Germany; Ospedale del Cuore G Pasquinucci Fondazione Toscana Gabriele Monasterio di Massa, ITALY

## Abstract

**Background:**

Soluble suppression of tumorogenicity 2 (sST2) has been shown to be of prognostic value in patients with chronic and acute left heart failure. The present study aims to assess the predictive value of sST2 levels in adult patients with complex congenital heart disease (CHD).

**Methods:**

In 169 consecutive patients with complex CHD and a mean age of 28.2 ± 12.0 years, sST2 levels were compared to 32 healthy controls and associated with clinical status as well as the occurrence of major adverse cardiac events (MACE). Mean follow-up time was 35.6 ± 24.9 months.

**Results:**

In CHD patients, median sST2 levels were 29.7 ng/ml compared to 26.4 ng/ml in healthy controls (p = 0.007) and increased with different types of CHD and the severity of MACE. According to ROC analysis, the most important predictors of acute heart/Fontan failure were NYHA class III/IV (AUC 0.804, p<0.001, CI 0.668–0.941), NT-proBNP levels (AUC 0.794, p<0.001, CI 0.640–0.948), γGT levels (AUC 0.793, p<0.001, CI 0.678–0.909) and sST2 levels (AUC 0.742, p = 0.004, CI 0.626–0.858), with NYHA class III/IV as the strongest independent predictor (p<0.001). All-cause mortality was best predicted by sST2 levels (AUC 0.890, p<0.001, CI 0.741–1.000), NT-proBNP levels (AUC 0.875, p = 0.001, CI 0.766–0.984) and NYHA class III/IV (AUC 0.837, p = 0.003, CI 0.655–1.000) with sST2 as the strongest independent predictor (p<0.001). Moreover, AUC increased to 0.918 combining both biomarkers and net reclassification improved with the addition of sST2.

**Conclusion:**

In patients with complex CHD, sST2 may have additive value to natriuretic peptides for the prediction of all-cause mortality.

## Introduction

The protein suppression of tumorogenicity 2 (ST2) is known to be involved in a wide range of pathophysiological processes. Before it was recognized to be involved in cardiovascular disease, it was mainly related to inflammatory and autoimmune disease [1]. It is expressed both in a transmembrane (ST2 ligand) and soluble form (sST2) with the latter blocking the cardioprotective effects of interleukin-33/ST2 ligand interaction and promoting myocardial apoptosis, fibrosis and hypertrophy [2,3]. Interleukin-33 and sST2 are expressed both in cardiac fibroblasts and cardiomyocytes and are induced either by biomechanical strain injury or angiotensin II [2]. Thus, expression levels are increased as a response to myocardial stress such a myocardial ischemia, mechanical overload or neurohormonal activation [4]. In cardiovascular disease, elevated sST2 levels maintained over time may indicate the presence of adverse myocardial remodeling and disease progression [5,6]. In the clinical setting, circulating sST2 levels have been shown to provide prognostic information in patients with acute and chronic left heart failure [7–13]. Moreover, serial measurement of sST2 concentrations may be useful for risk stratification and also predicting outcome in those patients [14–18]. In contrast to natriuretic peptides, sST2 levels seem not to be influenced by confounders such as age or renal function [17]. Additionally, the biologic and analytic variability of sST2 is much lower than that of natriuretic peptides suggesting that sST2 levels may be better in risk stratification and prognostication [19,20]. Thus, sST2 has been included in the ACC/AHA guidelines for additive risk stratification in patients with acute and chronic left heart failure [21].

Patients with complex CHD, namely those with a systemic morphological right ventricle or single ventricle physiology, are prone to develop acute heart/Fontan failure during long term follow-up [22,23]. However, risk stratification using natriuretic peptide levels is challenging in these patients due to the heterogeneity of the underlying lesions, different hemodynamics with univentricular or biventricular physiology and the presence of chronic hypoxia [24]. Nevertheless, natriuretic peptides have been proven to be of prognostic value in adult CHD patients [25].

To date, studies investigating sST2 levels in adult patients with complex CHD are lacking. According to the literature, there’s only one study available investigating sST2 levels in children and adolescents with CHD and dilated cardiomyopathy demonstrating a poor performance of sST2 in diagnosing pediatric heart failure [26]. However, the prognostic value of sST2 hasn’t been addressed in that study. Since sST2 has been proven to provide independent and incremental prognostic information in heart failure patients [9–11,14,27], the aim of our study was to measure sST2 concentrations and assess the predictive value of sST2 in adult patients with complex CHD.

## Materials and methods

### Patients

A total of 192 consecutive patients with complex CHD seen in our outpatient clinic between 22/12/2008 and 23/11/2016 were screened to be enrolled in the present study. Classification of CHD was performed according to current ACC/AHA guidelines [28] with complex CHD referring to as CHD of mostly great complexity such as all forms of pulmonary atresia, all forms of cyanotic CHD, Eisenmenger syndrome, transposition of the great arteries and all kinds of Fontan procedure as well as CHD at increased risk for heart failure such as tetralogy of Fallot, single ventricle physiology and transposition of the great arteries after atrial diversion procedure [23]. Exclusion criteria were other kinds of mild or moderate CHD, pregnancy, severe renal dysfunction or dialysis and incapability to understand or sign informed consent. Finally, 169 patients were included in this study of whom 61/169 (36.1%) patients had corrective surgery of congenital right heart disease (CRHD) such as pulmonary atresia or tetralogy of Fallot, 37/169 (21.9%) patients a systemic morphological right ventricle (SRV) of whom 10/37 patients presented with congenitally corrected transposition of the great arteries and 27/37 patients with d-transposition of the great arteries after atrial switch operation, 50/169 (29.6%) patients a single ventricle physiology after Fontan palliation (FONT) and 21/169 (12.4%) patients a non-corrected cyanotic heart defect or Eisenmenger physiology (EIS). Mean age was 28.2 ± 12.0 years (range 11–73 years). 84 patients were female and 85 patients male. Mean follow-up time was 35.6 ± 24.9 months with a median of 31 months. At each follow-up visit, a structured history and physical exam, a 12-lead surface electrocardiogram, measurement of blood pressure and transcutaneous oxygen saturation at rest and conventional two-dimensional echocardiography were performed in all patients. Follow-up investigations were made with special emphasis on clinical signs of pulmonary or systemic venous congestion including pleural effusions, ascites, peripheral edema with significant weight gain or protein losing enteropathy indicating acute heart/Fontan failure as well as the occurrence of supraventricular arrhythmias or atrial fibrillation. After echocardiography, venous blood was drawn for routine laboratory tests and blood sampling in all patients. Blood samples of patients were compared to those of 32 healthy subjects with a mean age of 29.6 ± 15.2 years (range 11–61 years) (S1 Dataset). The study complies with the Declaration of Helsinki, was approved by the Saarland medical association ethical board and all subjects or their guardians gave written and informed consent before enrollment.

### Conventional two-dimensional echocardiography

Two-dimensional echocardiography was performed using a Vivid 9 ultrasound system (GE Healthcare, Horten, Norway) with a transducer operating at 3.5 MHz. Apical 4-chamber views were acquired as loops for the planimetric calculation of the ejection fraction as well as endsystolic and enddiastolic volumes of the systemic ventricle according to the modified Simpson’s method [29]. Pulsed-wave Doppler echocardiography was performed to obtain the outflow pattern above the aortic valve to measure velocity time integral (VTI). Continuous-wave Doppler was used to estimate pressure gradients across the aortic or pulmonary valve. Colored Doppler flow was used to assess the severity of regurgitation across the systemic atrioventricular and aortic or pulmonary valve.

### Biochemical analyses

In all participants, venous blood samples were drawn into standard sampling tubes after echocardiography. Each sample was centrifuged and serum removed, allocated and frozen at -80°C before analysis of sST2 levels. In the patient group, routine laboratory tests such as liver function tests and creatinine measurements were performed using standard laboratory techniques. NT-proBNP levels were measured using an electrochemiluminescence sandwich immunoassay (Cobas^®^ proBNP II, Roche Diagnostics, Basel, Switzerland) on the Elecsys^®^ 2010 analyzer. Levels of sST2 were measured using a commercially available enzyme-linked immunosorbent assay (Presage^®^ST2, Critical Diagnostics, San Diego, California, USA) on the Sunrise^™^ Absorbance Microplate Reader (Tecan Trading AG, Mannedorf, Switzerland). All biochemical analyses were performed by investigators blinded to the clinical data of the patients. Glomerular filtration rate (GFR) was estimated using the chronic kidney disease epidemiology collaboration (CKD-EPI) creatinine equation [30].

### Data analysis

Clinical data of the patients were collected from medical records. The echocardiographic loops and Doppler images were stored digitally and analysed on an Echopac server (Echopac Version 6, GE Healthcare). Echocardiographic data sets were assessed by investigators blinded to the laboratory results. The presence of supraventricular arrhythmias or atrial fibrillation was confirmed either on the 12-lead surface or Holter electrocardiogram. Major adverse cardiac events (MACE) were defined as the occurrence of acute heart/Fontan failure or death from any cause. Patients were followed for fatal and non-fatal events until 31/12/2017.

### Statistical analysis

Data were analysed using standard statistical software (SPSS version 19; SPSS Inc., Chicago, Illinois). Continuous variables are expressed as mean ± standard deviation or median (interquartile interval) as appropriate. Differences between unpaired groups were analysed using a Mann-Whitney-U test for continuous variables and a chi-square test (or Fisher exact test, if numbers were small) for nominal variables. Correlations were evaluated using Spearman’s regression coefficient. For further analysis, log_10_ transformed biomarker values were used due to their skewed distribution. Receiver-operating characteristic (ROC) curve analysis was used for the prediction of acute heart/Fontan failure and all-cause mortality. Comparison of areas under the curve (AUCs) was performed using the DeLong method [31] and logistic regression analyses were used to assess combined biomarker models. Multivariate analysis was performed using Cox regression analysis to identify variables that independently predict MACE. Variables entered into the multivariate model were those that gave statistically significant results in the univariate analysis and didn’t show any multicollinearity. Furthermore, reclassification analysis was performed using net reclassification improvement (NRI) analysis [32]. A two-tailed p-value <0.05 was considered statistically significant.

## Results and discussion

### Results

#### Clinical data

During follow-up, a total of 28 adverse cardiac events occurred in 25/169 (13.0%) patients: 7/169 (4.1%) patients died (3 patients due to sudden cardiac death, 3 patients due to progressive heart/Fontan failure, 1 patient due to septicemia), 13 patients presented with acute heart/Fontan failure, and 8 patients had recurrent supraventricular arrhythmias or permanent atrial fibrillation indicating high atrial arrhythmic burden. Finally, 17/169 (10.0%) patients experienced MACE whereas 152/169 (90.0%) patients were in a clinically stable condition (S1 Dataset). Characteristics of patients with and without MACE are shown in Table 1.

**Table 1:** Characteristics of patients with and without MACE ^#^.

Variables	All patients (n = 169)	Patients without MACE (n = 152)	Patients with MACE (n = 17)	p-value*
Age at enrollment (years)	28.2 ± 12.0	28.3 ± 11.8	27.9 ± 13.9	0.780
Patients with Fontan palliation or Eisenmenger physiology (%)	71/169 (42.0%)	58/152 (38.2%)	13/17 (76.5%)	0.004
NYHA class	1.5 ± 0.7	1.4 ± 0.6	2.4 ± 0.8	< 0.001
Systolic blood pressure (mmHg)	120.3 ± 14.9	121.4 ± 14.4	111.4 ± 16.2	0.021
Diastolic blood pressure (mmHg)	70.1 ± 9.2	70.7 ± 9.1	65.1 ± 8.8	0.032
Transcutaneous oxygen saturation at rest (%)	94.7 ± 5.4	95.1 ± 4.9	91.7 ± 8.8	0.133
Ejection fraction of SV (%)	52.5 ± 10.8	53.3 ± 10.7	44.8 ± 8.3	< 0.001
Enddiastolic volume of SV (ml)	115.9 ± 58.5	113.4 ± 56.3	137.6 ± 74.0	0.136
Endsystolic volume of SV (ml)	58.0 ± 39.1	55.7 ± 37.9	78.0 ± 45.0	0.034
VTI above aortic valve (cm)	23.9 ± 4.4	24.1 ± 4.2	22.5 ± 5.7	0.292
Creatinine (mg/dl)	0.83 (0.70–0.95)	0.82 (0.70–0.94)	1.00 (0.58–1.34)	0.051
Estimated GFR (ml/min)	104.6 (91.4–117.7)	106.2 (93.4–119.0)	91.4 (68.3–107.5)	0.005
γGT (U/l)	38.0 (22.0–71.0)	34.0 (22.0–63.5)	78.0 (56.5–145.0)	<0.001
Albumin (g/l)	47.0 (44.0–49.0)	47.0 (45.0–49.0)	42.0 (39.5–49.0)	0.011
NT-proBNP (ng/l)	164.5 (81.5–436.2)	146.6 (74.6–330.4)	984.4 (383.5–1789.0)	< 0.001
sST2 (ng/ml)	29.9 (24.5–37.6)	29.4 (23.8–36.3)	39.8 (31.7–54.4)	< 0.001

MACE, major adverse cardiac event; NYHA, New York Heart Association; SV, systemic ventricle; VTI, velocity time integral; GFR, glomerular filtration rate

^#^ Mean ± standard deviation or median (interquartile interval) are used

* Patients with compared to those without MACE

#### Measurement of sST2 levels

**Fig 1:**
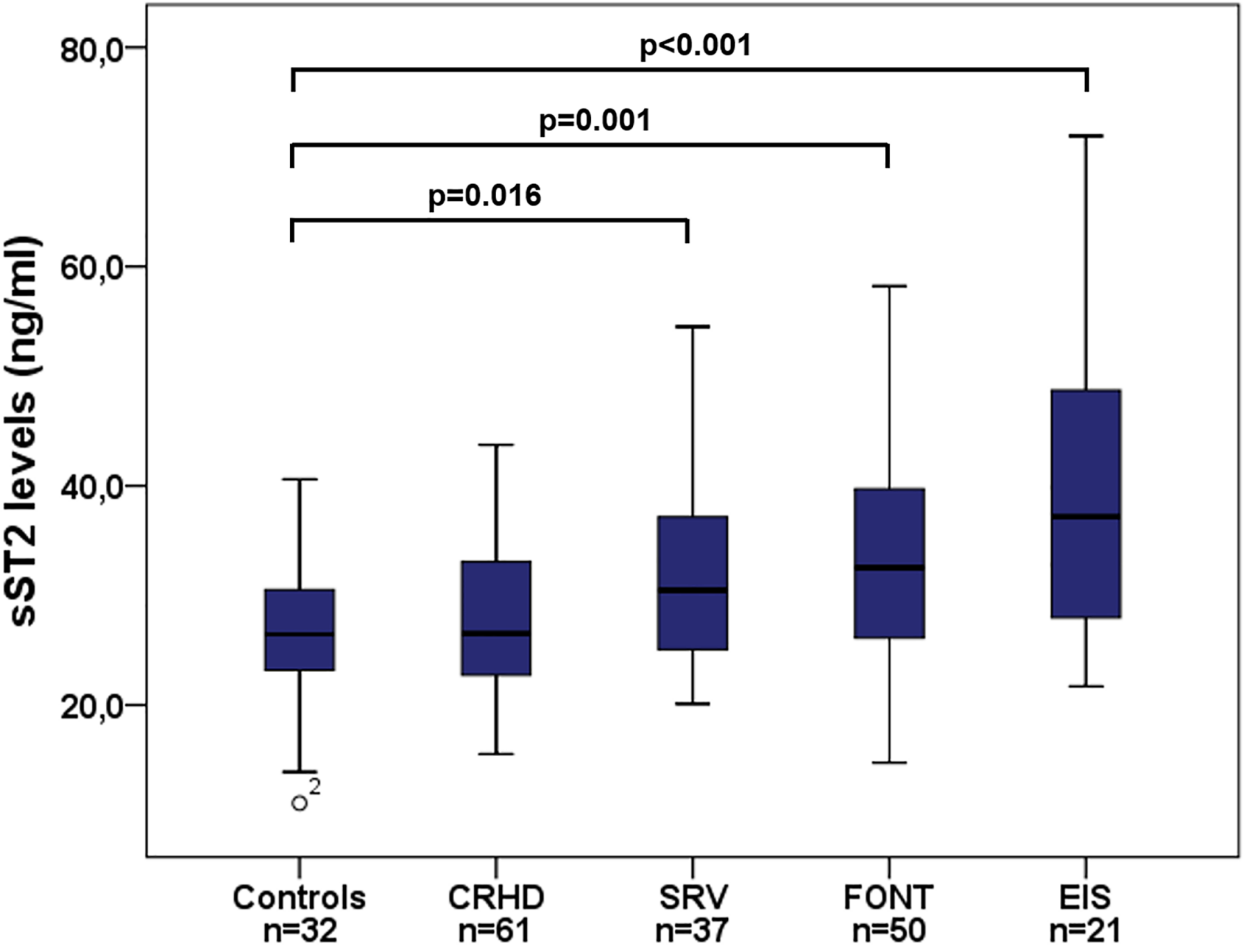
Boxplots displaying sST2 levels in healthy controls and patients with various types of congenital lesions. CRHD, corrected congenital right heart disease; SRV, systemic right ventricle; FONT, Fontan palliation; EIS, Eisenmenger physiology or unrepaired cyanotic heart defect.

sST2 levels ranged from 14.7 to 73.2 ng/ml with a median of 29.9 ng/ml (24.5–37.6 ng/ml) in the patient group and were significantly elevated as compared to healthy controls showing a median sST2 level of 26.4 ng/ml (23.0–30.5 ng/ml; p = 0.007). Significantly elevated sST2 levels were found in SRV patients with a median of 30.5 ng/ml (24.6–38.2 ng/ml; p = 0.016), in FONT patients with a median of 32.5 ng/ml (26.1–39.7 ng/ml; p = 0.001) and in EIS patients with a median of 37.2 ng/ml (26.7–49.1 ng/ml; p<0.001) (Fig 1) (S1 Dataset).

**Fig 2:**
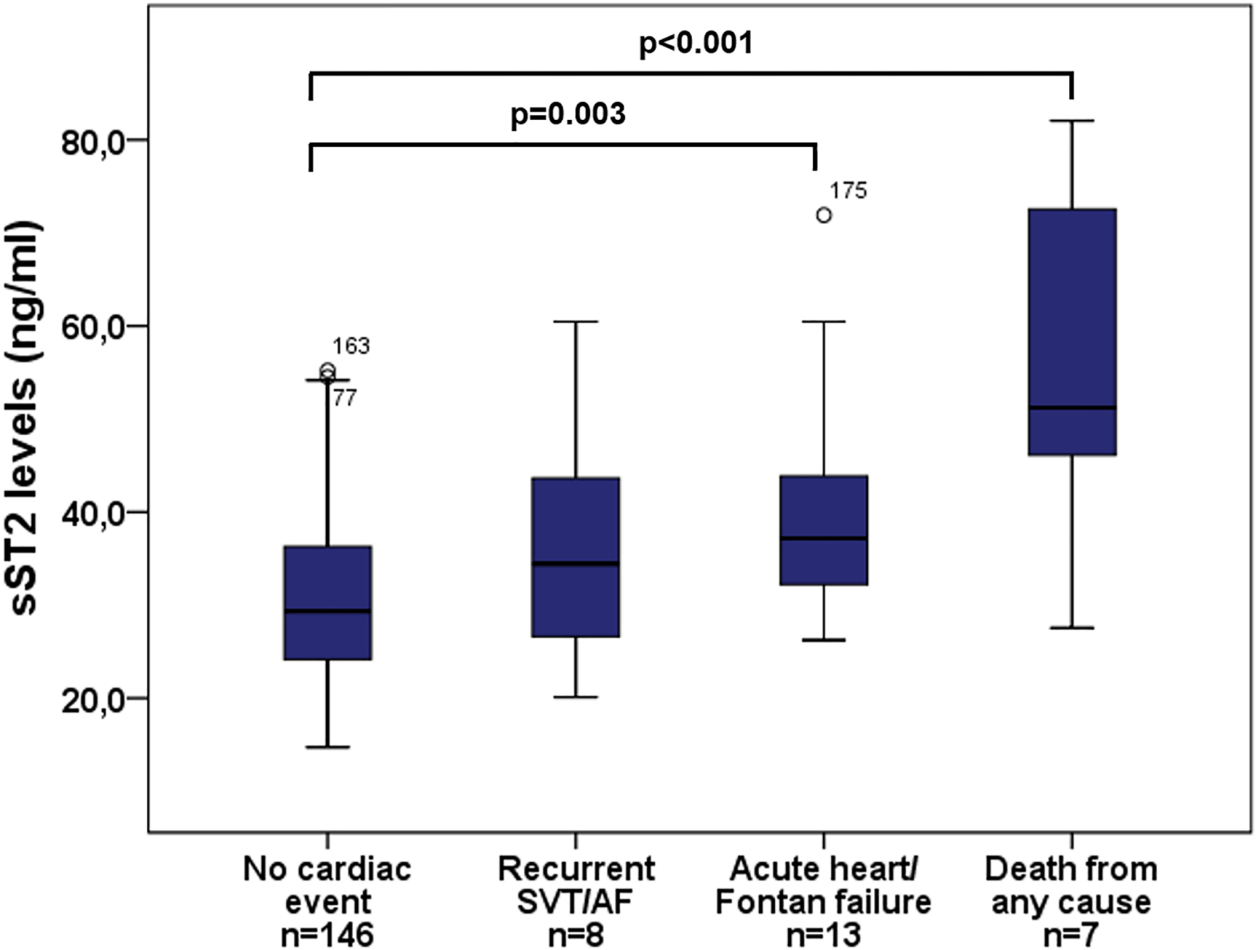
Boxplots demonstrating sST2 levels in patients with different adverse cardiac events. SVT, supraventricular tachycardia; AF, atrial fibrillation.

Median sST2 levels also increased according to MACE with highest levels found in deceased patients (Fig 2).

Levels of sST2 were only weakly related to NT-proBNP levels (r = 0.286; p<0.001). Relations of both biomarkers to clinical, echocardiographic and laboratory parameters are given in Table 2.

**Table 2:** Relation of sST2 and NT-proBNP with different variables*.

	sST2		NT-proBNP	
	r	p-value	r	p-value
Age at enrollment	0.094	0.208	0.440	< 0.001
NYHA class	0.192	0.013	0.552	< 0.001
Systolic blood pressure	0.126	0.103	-0.029	0.705
Diastolic blood pressure	0.115	0.136	0.013	0.865
Transcutaneous oxygen saturation at rest	-0.202	0.008	-0.180	0.019
Ejection fraction of SV	-0.178	0.020	-0.442	< 0.001
Enddiastolic volume of SV	0.020	0.798	0.045	0.560
Endsystolic volume of SV	0.017	0.829	0.166	0.031
VTI above aortic valve	-0.087	0.261	-0.273	< 0.001
Creatinine	0.243	0.001	0.153	0.047
Glomerular filtration rate	-0.160	0.038	-0.380	< 0.001
Albumin	-0.157	0.042	-0.299	< 0.001
γGT	0.241	0.002	0.249	0.001

sST2, soluble ST2; ns, not significant; NYHA, New York Heart Association; SV, systemic ventricle; VTI, velocity time integral

*Spearman rank correlation

#### Prediction of acute heart/Fontan failure

ROC curve analysis was used to identify predictors of acute heart/Fontan failure in all patients. The most significant predictors were a higher NYHA class III/IV, NT-proBNP levels, γGT levels and sST2 levels, respectively. Multivariate analysis identified a higher NYHA class III/IV as independent predictor of acute heart/Fontan failure (p<0.001) (Table 3).

**Table 3:** Results of ROC curve and multivariate analysis for the prediction of acute heart/Fontan failure and all-cause mortality.

**Prediction of acute heart/Fontan failure**				
Variables	AUC	95% CI	p-value	Multivariate analysis (p-value)
NYHA class III/IV	0.804	0.668–0.941	< 0.001	< 0.001
NT-proBNP	0.794	0.640–0.948	< 0.001	0.052
γGT	0.793	0.678–0.909	< 0.001	0.084
sST2	0.742	0.626–0.858	0.004	0.105
Ejection fraction of SV	0.713	0.601–0.824	0.011	0.863
GFR	0.682	0.518–0.846	0.030	0.116
Albumin	0.661	0.455–0.866	0.053	0.391
**Prediction of all-cause mortality**				
Variables	AUC	95% CI	p-value	Multivariate analysis (p-value)
sST2	0.890	0.741–1.0	< 0.001	< 0.001
NT-proBNP	0.875	0.766–0.984	0.001	0.687
NYHA class III/IV	0.837	0.655–1.0	0.003	0.054
GFR	0.810	0.670–0.950	0.006	0.716
Ejection fraction of SV	0.801	0.686–0.916	0.007	0.058
Systolic blood pressure	0.735	0.619–0.852	0.035	0.146
Creatinine	0.735	0.476–0.994	0.036	Not included

AUC, area under the curve; CI, confidence interval; NYHA, New York Heart Association; SV, systemic ventricle; GFR, glomerular filtration rate

**Fig 3:**
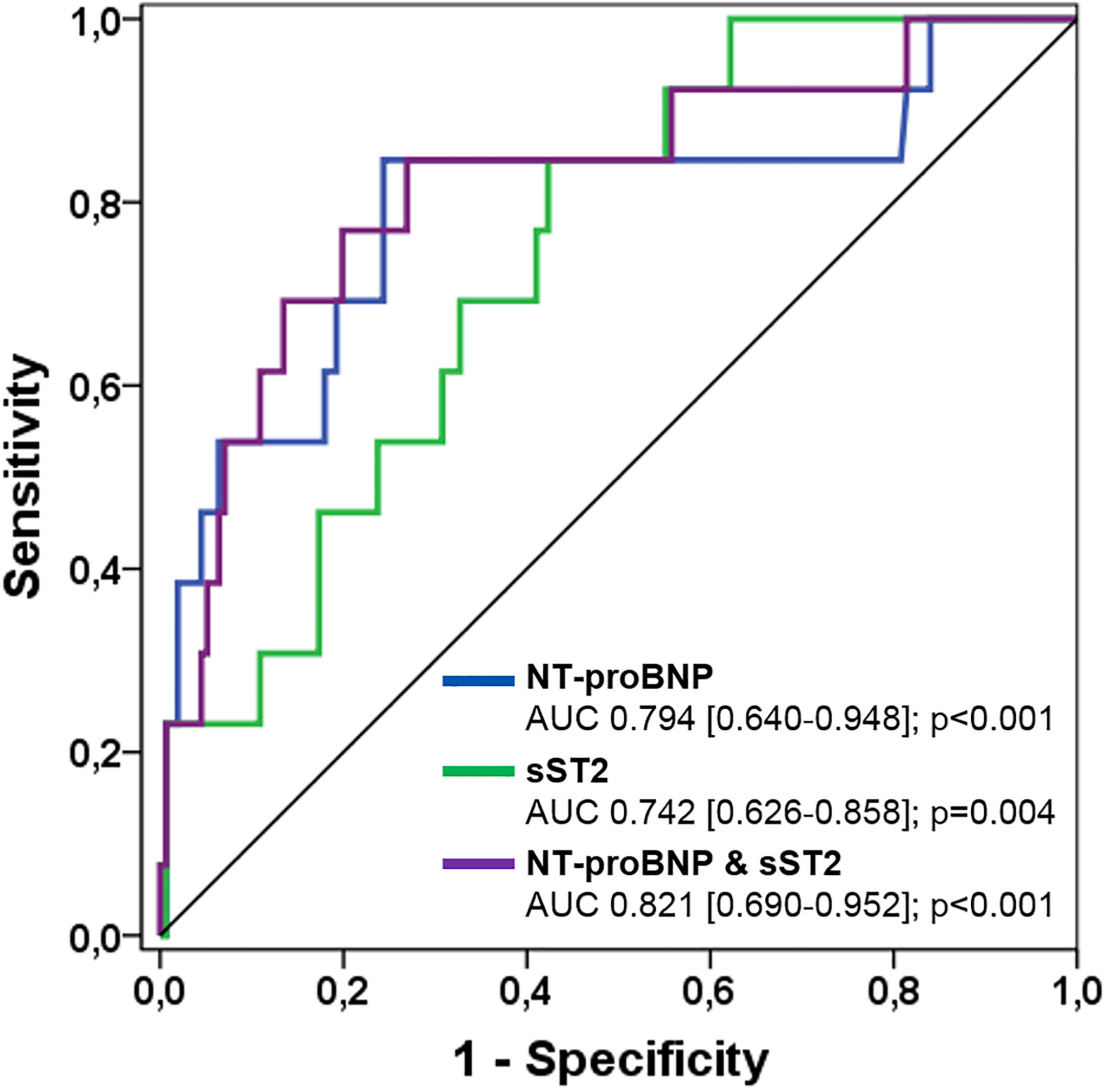
Receiver-operating characteristic (ROC) curves comparing sensitivity and specificity of sST2 and NT-proBNP levels in predicting acute heart/Fontan failure. AUC, area under the curve.

ROC curves of NT-proBNP and sST2 levels demonstrated no significant difference of AUCs of both biomarkers (p = 0.585), however, AUC increased slightly with the use of the combined biomarker model (Fig 3).

The optimal cut-off of sST2 levels for the prediction of acute heart/Fontan failure was calculated to be 31.1 ng/ml with a sensitivity of 84.6%, specificity of 58.3%, positive predictive value of 14.5% and negative predictive value of 97.8%. The optimal cut-off of NT-proBNP levels was calculated to be 349.5 ng/l with a sensitivity of 84.6%, specificity of 75.6%, positive predictive value of 22.4% and negative predictive value of 98.3%. NRI analysis was performed and components were calculated revealing an event NRI of 0% and a non-event NRI of -23.3% indicating no improvement of classification or risk prediction with the additional use of sST2.

#### Prediction of all-cause mortality

**Fig 4:**
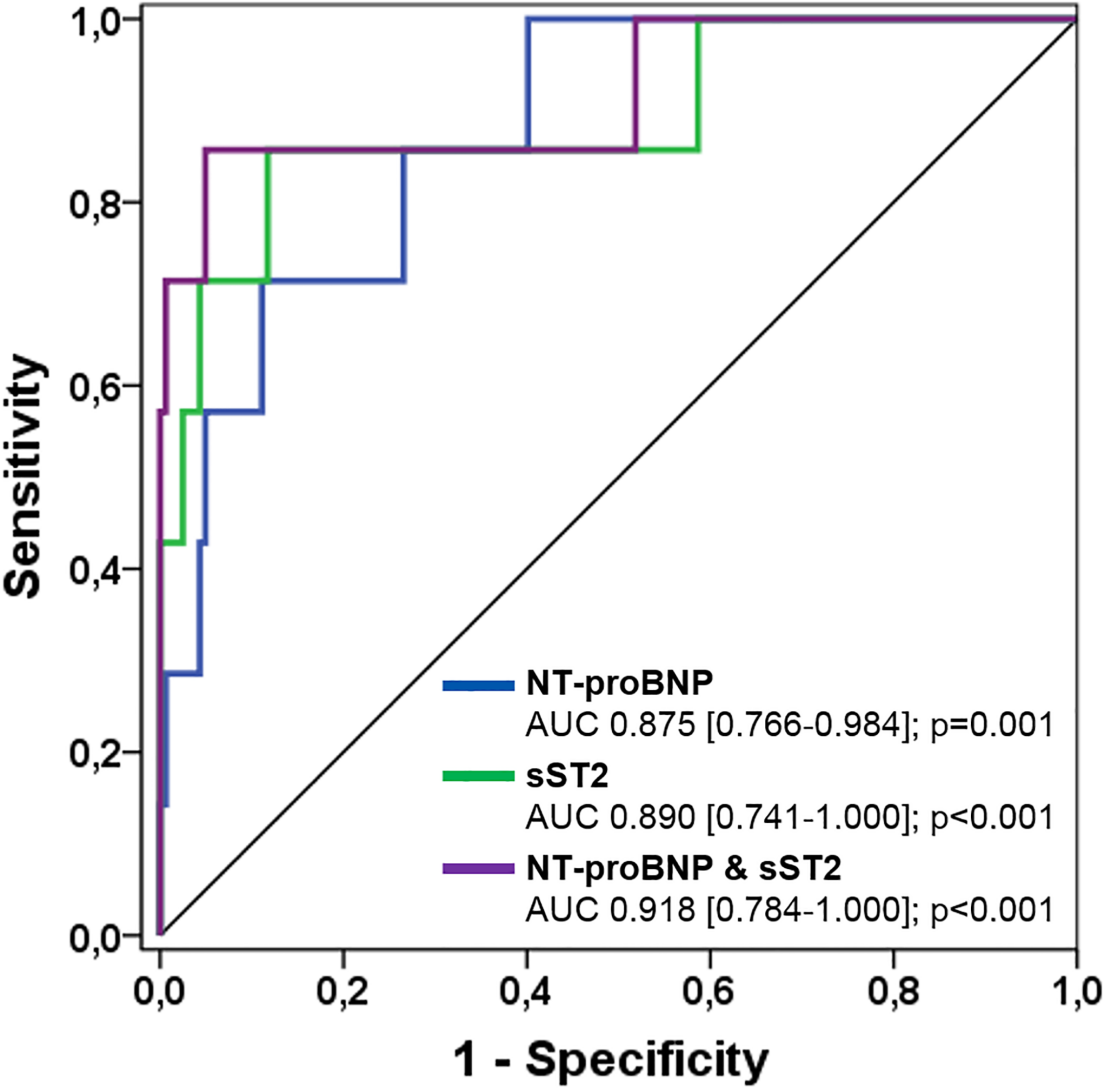
Receiver-operating characteristic (ROC) curves comparing sensitivity and specificity of sST2 and NT-proBNP levels in predicting all-cause mortality. AUC, area under the curve.

According to ROC curve analysis, the most significant predictors of all-cause mortality were sST2 levels, NT-proBNP levels, NYHA class III/IV, glomerular filtration rate and ejection fraction of the systemic ventricle, respectively. In the multivariate analysis, sST2 levels (p<0.001) turned out to be the most significant independent predictor of all-cause mortality (Table 3). Direct comparison of AUCs of NT-proBNP and sST2 levels revealed no significant difference (p = 0.788). However, AUC increased slightly using the combined biomarker model indicating an additive value of sST2 (Fig 4).

The optimal cut-off of sST2 levels for the prediction of all-cause mortality was calculated to be 42.8 ng/ml with a sensitivity of 85.7%, specificity of 88.3%, positive predictive value of 24.0% and negative predictive value of 99.3%. The optimal cut-off of NT-proBNP levels was calculated to be 774.6 ng/l with a sensitivity of 71.4%, specificity of 88.9%, positive predictive value of 21.7% and negative predictive value of 98.6%. Reclassification analysis using NRI showed an event NRI of 4.3% and a non-event NRI of -0.7% indicating that addition of sST2 improved classification and risk prediction of individuals with events, with almost no loss for non-events.

### Discussion

sST2 has emerged as a promising marker in patients with acute and chronic left heart failure providing incremental prognostic information and additional risk stratification to natriuretic peptides [10–14,18,27]. Thus, the aim of our study was to measure sST2 levels in patients with complex CHD and assess the predictive value of sST2 in this cohort of patients.

#### sST2 and its association with clinical status and adverse cardiac events

Our study demonstrates that sST2 levels are significantly elevated in the patient group as compared to healthy controls and increase with different types of CHD and the severity of MACE (Figs 1 and 2). Thus, highest sST2 levels were found in patients with Eisenmenger physiology and deceased patients being in agreement with previous studies that elevated sST2 levels are strongly associated with increased risk of death [8,9].

In our study, NT-proBNP but not sST2 was significantly related to measures of systolic ventricular function such as ejection fraction of the systemic ventricle and VTI above the aortic valve. Moreover, sST2 levels were not associated with high atrial arrhythmic burden reflecting altered loading conditions due to reduced systemic ventricular function or disease progression. Furthermore, sST2 and NT-proBNP levels were only slightly related to each other. Taken together, all these findings indicate a different pathophysiological mechanism of sST2 and NT-proBNP concentrations and thus a complementary or independent role of sST2.

#### Prediction of acute heart/Fontan failure

According to ROC curve analysis, the most significant predictors of acute heart/Fontan failure were a higher NYHA class III/IV, NT-proBNP, γGT and sST2 levels with an AUC of 0.804, 0.794, 0.792 and 0.742, respectively (Table 3). These findings are not surprising because NYHA class is known to be a strong and reliable predictor of acute heart failure and mortality in patients with chronic left heart failure and either reduced or preserved systolic ventricular function [33,34]. Natriuretic peptides are also well known for their prognostic capabilities in left heart failure but also CHD patients [21,25]. The predictive value of γGT levels in our study can be explained by the systemic venous congestion of the liver that is frequently present in patients with Fontan palliation or uncorrected complex heart defects without any palliation. sST2 was also found to be a significant predictor, however, didn’t show any improvement for the prediction of acute heart/Fontan failure using NRI analysis. Despite its subjective aspect of classification, a higher NYHA class III/IV turned out to be the strongest independent predictor in the multivariate analysis (p<0.001).

#### Prediction of all-cause mortality

The most important predictors of all-cause mortality in our cohort of patients were sST2, NT-proBNP, a higher NYHA class III/IV, glomerular filtration rate and ejection fraction of the systemic ventricle with an AUC of 0.890, 0.875, 0.837, 0.810 and 0.801, respectively. On multivariate analysis, sST2 turned out to be the strongest independent predictor of all-cause mortality (p<0.001). Moreover, ROC curve analysis showed an increase of AUC for the combined biomarker model. In addition, an improved classification of individuals with events was seen using NRI analysis thus underlining the potential additive value of sST2 for the prediction of death in CHD patients. Due to the small number of deceased patients in our study, however, these results should be validated in a larger cohort of CHD patients.

Nevertheless, our findings are in line with previous studies identifying sST2 as a robust prognostic marker in patients with left heart failure that may be due to the fact that sST2 is known to be a marker of myocardial fibrosis and inversed cardiac remodeling thus representing long-term cardiac alterations [2,4]. In contrast, natriuretic peptides respond to myocardial wall stretch and different loading conditions reflecting a completely different pathophysiological mechanism. The fact that sST2 has a lower biological and analytical variability [19,20] and is less influenced by confounders than natriuretic peptides [17] may also contribute to a better long-term prognostication.

#### Study limitations

This is a study that aims to assess the predictive value of sST2 in a high-risk subset of patients with complex CHD. Therefore, the period for patient recruitment is rather long and there is a wide range of ages in this cohort of patients in order not to miss patients with a failing Fontan circuit because Fontan failure can also occur at younger ages whereas acute heart failure in CRHD or SRV patients is more likely to occur in later adulthood. Nevertheless, it is surprising that the rate of MACE is rather low in this selected high-risk population thus indicating the need for a larger cohort of high-risk CHD patients and also a larger control group to be evaluated. Moreover, it would be interesting to know if serial sST2 measurements in CHD patients might be superior for prognostication than single measurements as has been shown in patients with left heart failure [16,18].

#### Conclusions

In patients with complex CHD, sST2 may have additive value to natriuretic peptides for the prediction of all-cause mortality and also is a strong independent predictor thus indicating its incremental prognostic value in this subset of patients.

## Supporting information

S1 Dataset Raw data of patients and controls. (XLSX)
